# Regulation of Mesenchymal Stem Cell Morphology Using Hydrogel Substrates with Tunable Topography and Photoswitchable Stiffness

**DOI:** 10.3390/polym14245338

**Published:** 2022-12-07

**Authors:** Samuel R. Moxon, David Richards, Oana Dobre, Lu Shin Wong, Joe Swift, Stephen M. Richardson

**Affiliations:** 1 Division of Cell Matrix Biology and Regenerative Medicine, School of Biological Sciences, Faculty of Biology, Medicine and Health, Manchester Academic Health Science Centre, University of Manchester, Manchester M13 9PL, UK; 2 The Henry Royce Institute, University of Manchester, Manchester M13 9PL, UK; 3 Centre for the Cellular Microenvironment, Advanced Research Centre, University of Glasgow, Glasgow G12 8LT, UK; 4 Manchester Institute of Biotechnology and School of Chemistry, University of Manchester, 131 Princess Street, Manchester M1 7DN, UK; 5 Wellcome Centre for Cell-Matrix Research, University of Manchester, Oxford Road, Manchester M13 9PT, UK

**Keywords:** mechanosensing, mechanotransduction, hydrogel, mesenchymal stem cell

## Abstract

Cell function can be directly influenced by the mechanical and structural properties of the extracellular environment. In particular, cell morphology and phenotype can be regulated via the modulation of both the stiffness and surface topography of cell culture substrates. Previous studies have highlighted the ability to design cell culture substrates to optimise cell function. Many such examples, however, employ photo-crosslinkable polymers with a terminal stiffness or surface profile. This study presents a system of polyacrylamide hydrogels, where the surface topography can be tailored and the matrix stiffness can be altered in situ with photoirradiation. The process allows for the temporal regulation of the extracellular environment. Specifically, the surface topography can be tailored via reticulation parameters to include creased features with control over the periodicity, length and branching. The matrix stiffness can also be dynamically tuned via exposure to an appropriate dosage and wavelength of light, thus, allowing for the temporal regulation of the extracellular environment. When cultured on the surface of the hydrogels, the morphology and alignment of immortalised human mesenchymal stem cells can be directly influenced through the tailoring of surface creases, while cell size can be altered via changes in matrix stiffness. This system offers a new platform to study cellular mechanosensing and the influence of extracellular cues on cell phenotype and function.

## 1. Introduction

Understanding how the native extracellular environment influences cell shape and subsequent function is valuable in the design of in vitro culture systems for the recapitulation of in vivo cell phenotypes. Cell morphology has been shown to play an integral role in the function of many cell types [1]. In cartilaginous tissues, for example, a spherical cell morphology has been repeatedly demonstrated to be critical for extracellular matrix (ECM) homeostasis, while the transition into a ‘fibroblastic’ morphology has been linked to the loss of phenotype and the production of a compromised matrix [2,3]. Conversely, myoblasts rely on the formation of an aligned and elongated morphology in order to facilitate the formation of myotubes and the subsequent functional contractility of myofibrils [4]. The precise control of cell morphology and size, however, continues to present a challenge in the design of in vitro culture systems. Multiple studies have reported an inability to regulate cellular mechanisms as a direct result of uncontrollable changes in cell shape [2,5,6]. To address this issue, there has been increasing focus on tailoring features such as the surface topography, chemistry and stiffness of cell culture substrates.

Multiple studies have, for example, demonstrated that alterations in substrate surface topography and/or stiffness can influence the adhesion, proliferation, morphology and alignment of mesenchymal stem cells (MSCs) and induced pluripotent stem cells [7,8,9,10,11,12,13]. In particular, concave surface features, such as grooves and trenches, have been shown to directly influence cell morphology and directionality [14,15], while an increase in matrix stiffness has been evidenced as a contributing factor in increased cell spreading and size [13]. The exact mechanisms by which surface properties affect overall cell structure have not been fully elucidated, but it is proposed that surface topography and stiffness can directly regulate the structuring of the actin cytoskeleton and alter cellular behaviour via biochemical and mechanical signalling pathways [16,17,18].

The influence of cell substrate stiffness and topography on MSC behaviour is, therefore, well-evidenced in the literature. However, the exploitation of these cell–material interactions for technological applications has, thus far, been largely limited to cell culture materials and surfaces with properties that are static (i.e., fixed at the point of production), thus, eliminating any ability to temporally regulate the extracellular environment in situ. More recently, interest in the field has shifted towards systems that can allow for more dynamic control over the cell morphology and phenotype with the temporal control of extracellular factors such as matrix stiffness [19,20] or surface topography [21], but there is still a lack of systems that allow for such a manipulation of structural and mechanical properties in parallel. More accessible platforms for controlling both stiffness and topography are, therefore, required to better understand the interplay between them, particularly whether they can be controlled in a dynamic (i.e., time-dependent) fashion. Additionally, systems that allow for the regulation of the mechanical and structural properties of the cell culture substrate in real time are of increasing value, because they allow for the observation of dynamic cellular responses to changes in the extracellular environment. We have previously demonstrated that the stiffness of polyacrylamide hydrogels bearing photoisomerisable azobenzene crosslinks can be manipulated using irradiation with the appropriate wavelength of light, and that azobenzene-crosslinked polyacrylamide (AZO-PA) hydrogels can be manipulated in real-time to temporally control the morphology of human MSCs [19,22].

This study extends the application of this phototunable hydrogel platform, through the introduction of controllable surface topography, to demonstrate a simple method for controlling the morphology and size of human MSCs. We demonstrate that reticulation, produced via a ‘sandwich layer’ method of hydrogel casting reported herein, facilitates the engineering of creased topographic features on the surface of the gel [23]. The periodicity, branching and length of these features can be easily tailored via the modulation of the gel thickness. Moreover, in this investigation, MSC behaviour was successfully regulated via a combination of the dynamic matrix stiffness and a newly presented tuneable surface topography. The design of a system for directly influencing cell shape and size, thus, provides a platform for better understanding of cell–substrate interactions, and could aid the design of materials that can better regulate cell behaviour and provide a greater understanding of cell–biomaterial interactions.

## 2. Materials and Methods

### 2.1. Materials

All reagents used in this study were sourced from standard suppliers, unless otherwise stated, and used without further purification. Alexa Fluor488-phalloidin was purchased from Cell Signaling Technology (Danvers, MA, USA). For the cell culture experiments, the hydrogels were cast between 18 mm glass coverslips and 35 mm diameter glass-bottomed dishes (uncoated, γ-irradiated, MatTek Corp, Ashland, MA, USA). The Y201 immortalised MSCs were a kind gift from Prof Paul Genever (University of York, York, UK) that were generated via a previously published method [24], with which we previously demonstrated to possess similar mechanoresponsive properties to primary human MSCs [25]. All experiments that utilised cell culture media were conducted using Dulbecco’s modified Eagle’s medium (DMEM, 1 g/L glucose, L-glutamine, sodium pyruvate, phenol red, without HEPES) supplemented with 10% (*v*/*v*) foetal bovine serum (FBS) and an antibiotic/antimycotic solution (100 units/mL penicillin, 100 µg/mL streptomycin, 0.25 µg/mL amphotericin).

### 2.2. Synthesis of AZO Crosslinked Polyacrylamide Hydrogels

AZO-PA hydrogels were prepared using a previously reported method [22]. Briefly, 4,4′-di(acrylamido)azobenzene (AZO) was dissolved in dimethylsulfoxide (DMSO), and aqueous solutions of acrylamide (AM, 40% *w*/*v*) and N,N′-methylenebisacrylamide (BIS, 0.1 M) were mixed with phosphate-buffered saline (PBS). The AZO and AM/BIS solutions were, subsequently, mixed with 10% ammonium persulfate (*w*/*v* in water, 10 μL) and N,N,N′,N′-tetramethylethylenediamine (1 μL). These mixtures were polymerised on glass-bottomed Petri dishes for 30 min at room temperature prior to the analysis. In order to prepare hydrogels with varying crease topography densities, prepolymer solutions with varying volumes were placed in the dishes (4, 6 or 8 µL; corresponding to a final hydrogel disc thicknesses of 50, 75 and 100 µm, respectively). Glass coverslips were then placed on top of the droplets to generate hydrogel discs with thicknesses of 50 µm, 75 µm and 100 µm via the sandwich layer mechanism in the glass-bottomed dishes [22]. All other parameters remained constant in order to ensure that the disc thickness was regulated solely by droplet volume. The samples were then washed with PBS to remove any unpolymerised materials and DMSO. The fabricated hydrogels were, subsequently, immersed in phenol red-free media in an incubator at 37 °C, 5% CO_2_, for 48 h prior to further use.

### 2.3. Characterisation of Topographic Density

The surfaces of AZO-PA hydrogels were analysed using an EVOS bright-field microscope (ThermoFisher, West Sussex, UK). Images were taken in phase contrast mode and, subsequently, processed and analysed using Fiji/ImageJ version 1.52p. Images were converted to greyscale before either being inverted or subjected to background subtraction and binarization (using Huang thresholding [26]; validity determined via a raw image comparison). Further characterisation was conducted using the fast Fourier transform (FFT) tool. Three binary images (1024 × 1024 pixels each) from different areas across the surface of each hydrogel sample were recorded and analysed. Additionally, the ImageJ measuring tool was used to determine the mean crease length for each gel thickness (n = 3 gel samples for each thickness). The degree of branching in the creased topography was also determined for each gel thickness using ImageJ and standardised to branch points per 0.05 mm^2^ using the area tool.

Atomic force microscopy (AFM) was employed to generate height maps of the topographical surface features of the hydrogels. Samples were analysed using a Bruker Bioscope Catalyst AFM (Bruker, Coventry, UK). Imaging was performed in phenol red-free Dulbecco’s modified Eagle’s medium using cantilevers with 5 μm diameter spherical glass probes (Bruker, Coventry, UK). Scans of topographical features were recorded at 5 randomly selected 50 μm × 50 μm areas of the hydrogel surface, with a sampling rate of 96 height measurements/scan line and a scan rate of 1 Hz.

### 2.4. Modulation of Matrix Stiffness and Cell Seeding

Hydrogel stiffness was controlled with photoirradiation with LED light sources according to previously reported methods [22]. To generate the initial low-stiffness gels, the gels were irradiated for 30 min at 365 nm, allowed to rest in a dark room under ambient conditions for 2.5 h and then incubated at 37 °C, 5% CO_2_, for 21 h in the dark. The hydrogels used for the negative controls were subjected to the same conditions, except that the photoexposure was omitted. Soft ‘(−) blue’ samples were, subsequently, generated through a further incubation of 24 h, while stiff ‘(+) blue’ samples were generated using a 60 min irradiation at 490 nm before a 23 h incubation at 37 °C, 5% CO_2_. The substrate stiffness was, subsequently, determined with AFM using the same procedure as in Section 2.3.

For cell-based analyses, MSCs were seeded on to the hydrogels at a density of either 5000 or 500 cells/cm^2^ following an initial low-stiffness gel formation (irradiation for 30 min at 365 nm, followed by 2.5 h in the dark). For the evaluation of the effect of surface topography on cell size and morphology, cells were cultured for 48 h without the further processing of the gels post cell seeding.

To assess the effect of substrate stiffness on the cell size and morphology, cell-laden gel stiffness was modified via photoirradiation. Cell-laden samples were either cultured in the dark for 48 h for soft ‘(−) blue’ samples, or cultured in the dark for 24 h, then irradiated at 490 nm for 60 min and cultured in the dark for a further 24 h for the stiffened ‘(+) blue’ samples. Negative control samples were seeded on nonirradiated stiff hydrogels and cultured in the dark for 48 h.

### 2.5. Analysis of Cell Morphology and Size

At the end of the culture periods for all cell-based analyses, the cells were fixed in 10% paraformaldehyde (PFA), washed three times with PBS and permeabilised in 0.25% triton-X for 5 min. Samples were then washed in Dulbecco’s modified phosphate-buffered saline (DPBS) three times, blocked for 30 min in 10% BSA and incubated in a solution of DAPI (1:2000) and AF488-labelled phalloidin (1:100) for 30 min (room temperature, no visible light). Cytoskeletal imaging was, subsequently, conducted using an Olympus BX51 upright microscope with DAPI and FITC filters. To visualise cell spreading along surface creases, a combination of fluorescence and correlative bright-field imaging was also employed with a 10×/0.30 UPlanFLN objective.

In order to quantitatively assess the influence of hydrogel surface topography on cell morphology and size, fluorescent images of seeded MSCs were processed in ImageJ v1.52a. CellProfiler v4.2.0 image analysis software was used to determine cell and nuclear alignment, area, eccentricity and solidarity. Additionally, merged fluorescence and phase contrast images were used to discern between cells adhered to the surface topography and cells attached to the gaps between the topographies. The collected data were statistically analysed via the Mann–Whitney test, with *p* < 0.05 representing the statistical significance.

### 2.6. Live Cell Imaging

Time-lapse images of MSCs were captured 24–48 h after the initial seeding via phase contrast and fluorescence microscopy (as in Section 2.5). Samples were imaged under phenol red-free media at 37 °C and 5% CO_2_. Point visiting and laser-based autofocus (Perfect focus, Nikon) were used to allow for repeated imaging of multiple positions throughout a continuous time course. Images were collected every 30 min with consistent exposure times using NIS Elements AR.46.00.0 imaging software and a Retiga R6 (Q-Imaging) camera. Time-lapse images were, subsequently, processed and analysed using Fiji v1.0.0 and CellProfiler v4.2.0 software.

## 3. Results

### 3.1. Characterisation of Hydrogel Surface Topography

**Figure 1:**
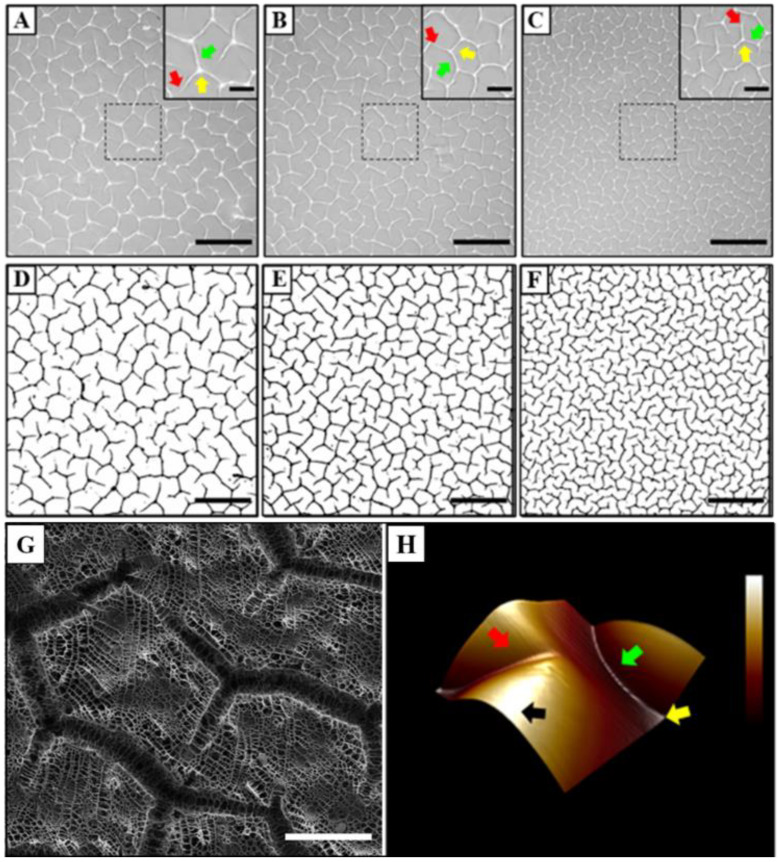
Topographical features of AZO-PA hydrogels. Phase-contrast images of creases on hydrogel surfaces formed with (**A**) ~100, (**B**) ~75 and (**C**) ~50 μm gel thickness (scale bar = 500 µm); binarized images of topographies on (**D**) ~100, (**E**) ~75 and (**F**) ~50 μm gel thickness (scale bar = 500 µm); (**G**) SEM image highlighting the surface creases at high magnification (scale bar = 250 µm); and (**H**) a 3D AFM map of the gel surface topography. Arrows indicate morphological components (red = disconnected branch; yellow = junction; and green = connected branch) and approximate force curve recording position (black) at convex surface region amenable to probe engagement.

Initially, a systematic characterisation of the hydrogels was performed under control conditions. Here, AZO-PA hydrogel discs of 50, 75 or 100 μm thickness were formed on Petri dishes using the sandwich layer method and imaged with phase-contrast microscopy. It was observed that a network of creases formed on the surface in a uniform manner, with thinner gels producing a higher density of creases than thicker gels (Figure 1A–H). To more accurately visualise the topology of these features, SEM and AFM imaging were performed on the gels, which revealed a network of three-dimensional cavities that covered the entire surface area of the gel (Figure 1G,H).

**Figure 2:**
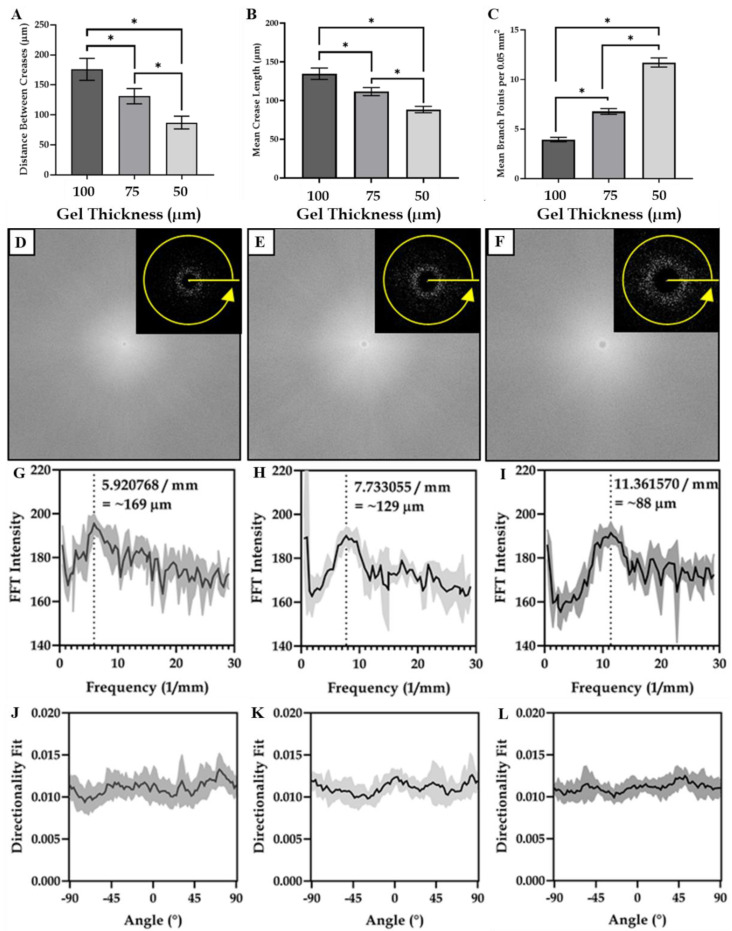
Regulation of hydrogel topography via gel thickness. (**A**) The average distance between surface creases plotted as a function of gel thickness (* indicates *p* < 0.05); (**B**) the mean length of the surface creases as a function of topography type; (**C**) the mean branch points of the surface creases per 0.05 mm^2^ as a function of topography type; 2D-FFTs for (**D**) ~100, (**E**) ~75 and (**F**) ~50 μm gel thickness; FFT intensity for (**G**) ~100, (**H**) ~75 and (**I**) ~50 μm gel thickness and the directionality fit for (**J**) ~100, (**K**) ~75 and (**L**) ~50 μm gel thickness.

A more detailed analysis of the microscopy images revealed that the distance between the creases and crease length both decreased as the gel thickness decreased, while the number of branch points increased (Figure 2A–L). A two-dimensional Fourier transform (2D-FFT) of the images indicated an inverse relationship between the gel layer thickness and periodicity distance (average distance between the neighbouring creases—Figure 2D–I), with gels formed with a ~50 µm gel thickness exhibiting the highest topographical densities (creases per unit area), as evidenced by the lowest distances between the surface creases. No difference in crease directionality was identified between any of the surfaces (Figure 2J–L).

### 3.2. Adhesion and Migration Analysis of MSCs

**Figure 3:**
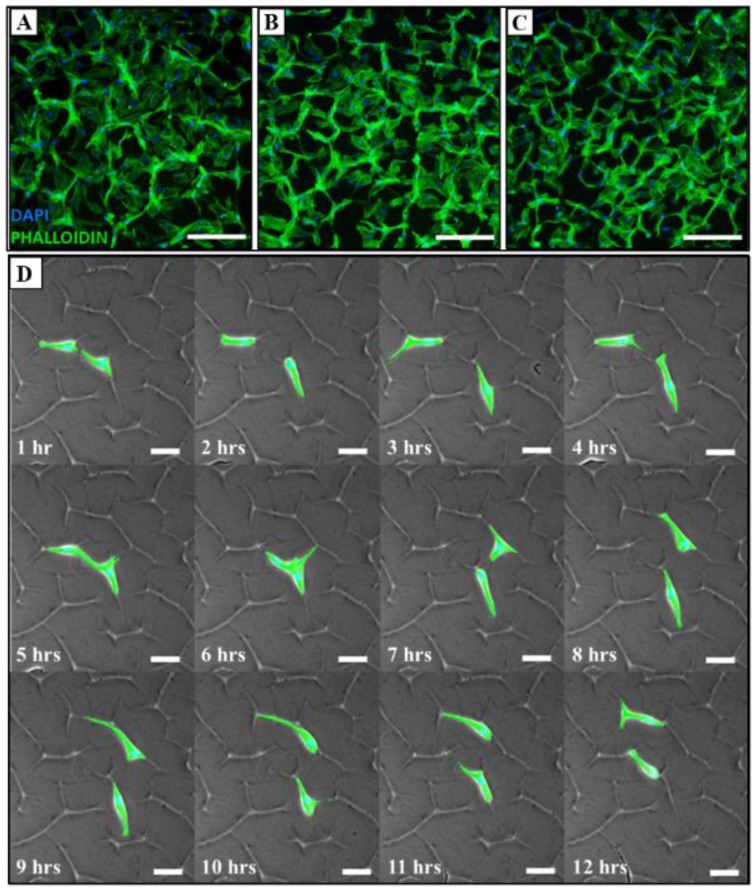
MSCs adhered, spread and migrated along surface topographies. DAPI/phalloidin of MSCs seeded at 5000 cells/cm^2^ on AZO-PA hydrogels with (**A**) ~100, (**B**) ~75 and (**C**) ~50 μm gel thickness (scale bar = 250 µm) and (**D**) sequential images from live cell tracking demonstrating MSC migration along surface creases over 12 h for cells seeded at 500 cells/cm^2^ (scale bar = 50 µm).

MSCs were then cultured on hydrogels for each of the three corresponding thicknesses for 48 h. The fluorescence imaging of DAPI/phalloidin-labelled MSCs revealed that the cells preferred to occupy the hydrogel creases in a density-dependent manner. More specifically, when seeded at a density of 5000 cells/cm^2^, the cells attached to the surface topographies and spread across the spaces between them (Figure 3A–C). However, when seeded at a lower density of 500 cells/cm^2^, it was found that the cells favoured adhering to the surface creases (Figure 3D). Moreover, once adhered to these surface features, the cells preferentially migrated along them, and did not relocate to the spaces between the surface features (Figure 3D). These lower seeding densities were, thus, used in all subsequent experiments.

### 3.3. Modulating Cell Morphology and Alignment via Tuneable Surface Topographies

**Figure 4:**
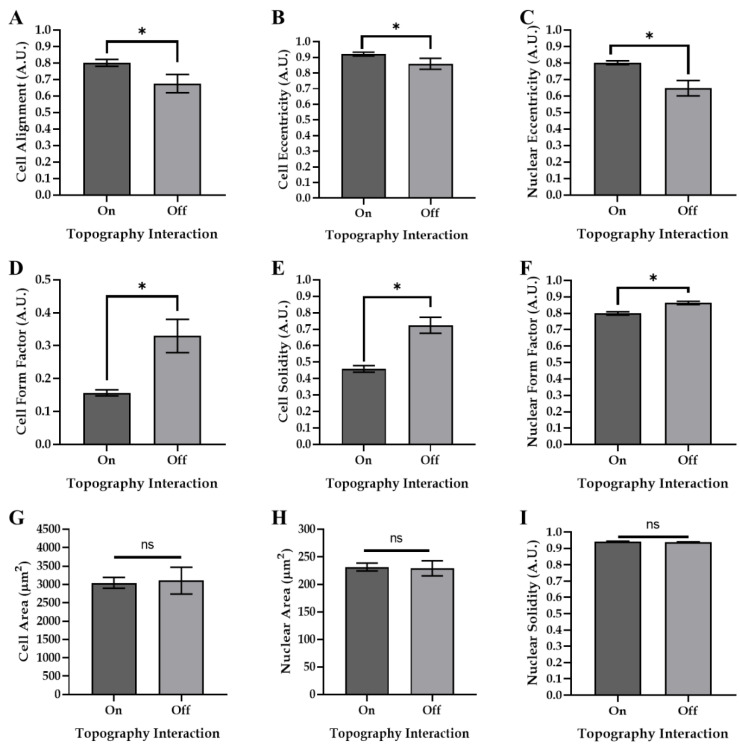
Adhesion to the creased topography influenced cell morphology but not cell size. The impact of cell adherence on and off the creased topographies on (**A**) cell alignment, (**B**) cell eccentricity, (**C**) nuclear eccentricity, (**D**) cell form factor, (**E**) cell solidity, (**F**) nuclear form factor, (**G**) cell area, (**H**) nuclear area and (**I**) nuclear solidity. (‘On’—cells in contact with topographical features/creases; ‘Off’—cells not in contact with topographical features. * indicates *p* < 0.05; ns indicates no significant differences.

**Figure 5:**
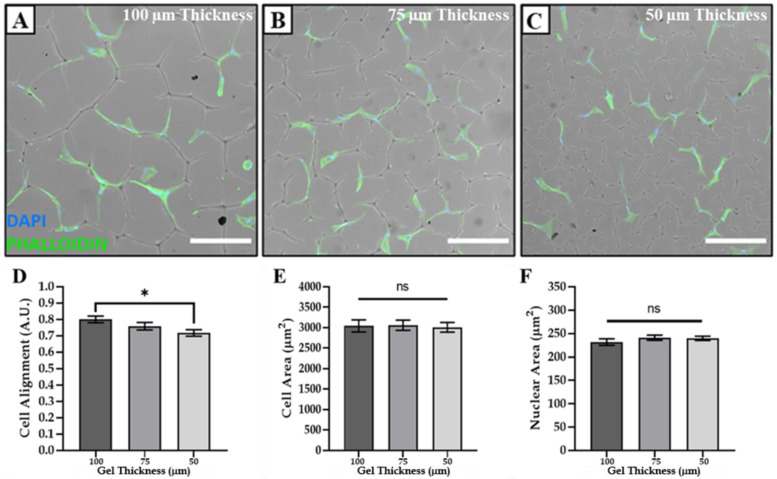
Cell alignment could be tailored by surface topography without affecting cell size. DAPI/phalloidin of MSCs seeded at 500 cells/cm^2^ on AZO-PA hydrogels with (**A**) ~100, (**B**) ~75 and (**C**) ~50 μm gel thickness (scale bar = 250 µm) and the effect of topography type/periodicity distance on (**D**) cell alignment, (**E**) cell area and (**F**) nuclear area. * indicates *p* < 0.05; ns indicates no significant differences.

In order to study how the cell shape was affected by contact with the surface creases, phalloidin staining of adhered Y201 MSCs was carried out to enable the detailed image analysis of their size and morphology. This analysis showed that the cell shape was directly influenced by adherence to the surface creases, as indicated by an increase in cell/nuclear alignment and eccentricity on the topography compared to cells that were not in contact with the topographical features, and a reduction in the cell/nuclear form factor and solidity (Figure 4A–F). Interestingly, the cell size was not affected by adhesion on the surface creases (Figure 4G–I), as no significant differences in cell area were observed between those cells that were located on the creases of the gel (on—Figure 4) compared to those that were on the flat areas of the gel surface (off—Figure 4). However, the extent of the cell alignment was found to be influenced by the topographical density of the surface creases (Figure 5D). The alignment was inversely correlated to the topographic density, with cells exhibiting the highest level of alignment on AZO-PA hydrogel surfaces exhibiting the lowest topographic density (i.e., highest periodicity distance; Figure 5A–D). Cell and nuclear sizes were not affected by topographic density (Figure 5E,F).

### 3.4. Tailoring Matrix Stiffness and Cell Size

**Figure 6:**
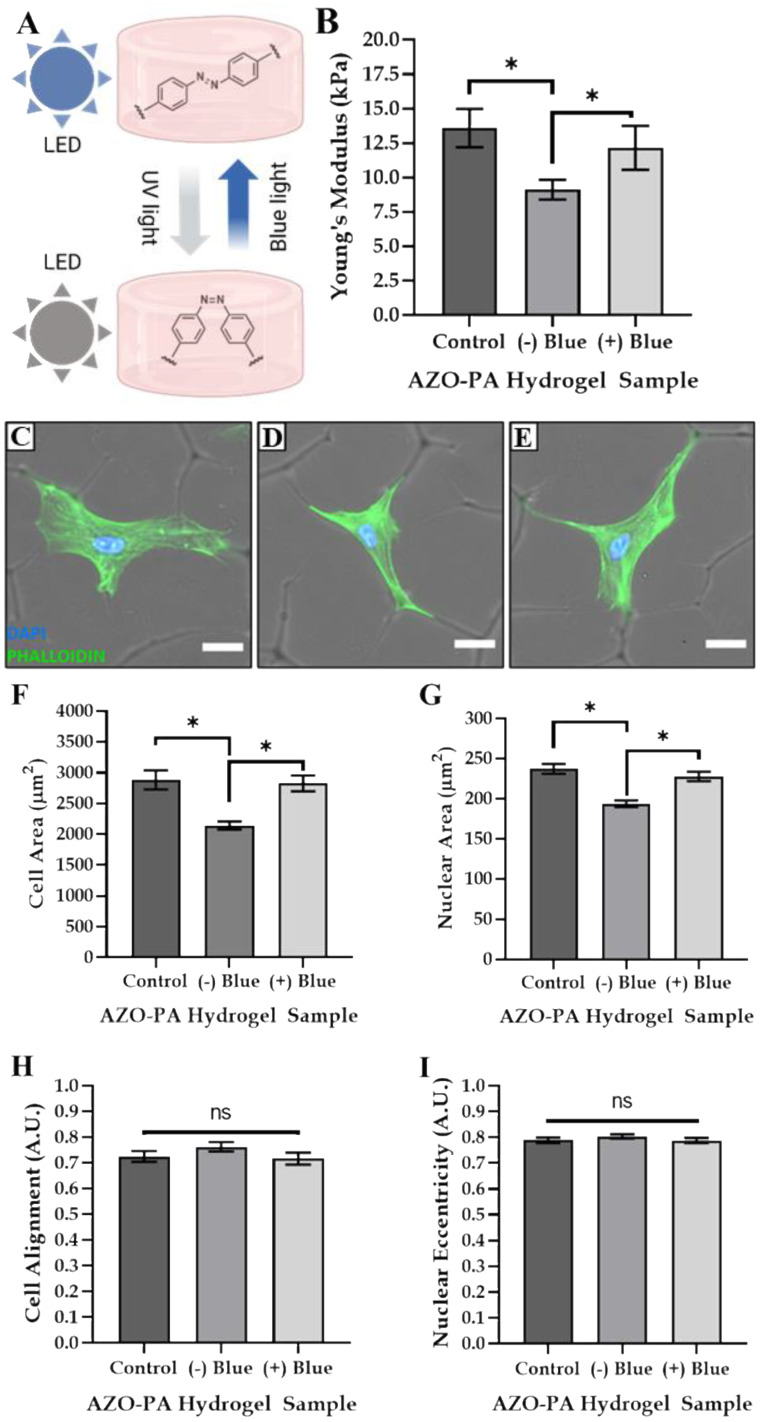
Photoswitchable matrix stiffness influenced cell size but not cell shape. (**A**) A schematic, created with BioRender.com, demonstrating the mechanical tuning of AZO-PA hydrogels with light; (**B**) the influence of photo-crosslinking method on the Young’s modulus of AZO-PA hydrogels; DAPI/phalloidin staining of MSCs cultured on (**C**) control, (**D**) (−) blue and (**E**) (+) blue hydrogel samples (scale bar = 25 µm) and the effect of matrix stiffness on (**F**) cell area, (**G**) nuclear area, (**H**) cell alignment and (**I**) nuclear eccentricity. * indicates *p* < 0.05, ns indicates no significant differences.

The photomodulation of the AZO-PA hydrogels was performed as per the methods detailed in Section 2.4 to investigate the effect of tailoring the substrate stiffness in situ on the cell size and shape of MSCs. The mechanical properties of the hydrogels were successfully tailored via a previously reported method of irradiation [22] (Figure 6A,B). Firstly, a hydrogel thickness of 50 µm with the most densely reticulated surface was used to investigate the influence of matrix stiffness in addition to the presence of topographic features. Fluorescence images of DAPI/phalloidin-stained MSCs demonstrated that tuning the matrix stiffness did not inhibit the preferential cell adhesion to the surface topographies (Figure 6C–E). Tailoring the matrix stiffness also had no impact on cell alignment, eccentricity or other morphological factors, but cell size was found to be directly influenced by the mechanical properties of the hydrogel (Figure 6F–I). More specifically, a reduction in substrate stiffness significantly reduced both cell and nuclear areas with no impact on cell shape.

## 4. Discussion

This study presented a simple method for independently regulating cell size and morphology by tailoring the matrix stiffness and surface features of AZO-PA hydrogels. The sandwich layer technique reported herein enabled the preparation of reticulated hydrogels with surface creases. The surface density of these creases could be altered by changing the pregel polymerisation volume, thereby tailoring the gel thickness (Figure 1). The subsequent formation of these surface creases was likely a result of gel shrinkage upon the removal of the organic solvent during the reticulation process. It was found that the periodicity (and, therefore, the feature density) of the creases was customisable as a function of gel thickness, with a higher topographical density corresponding to a reduced layer thickness. It was also observed that the gels that formed with lower gel thicknesses appeared to exhibit a network of creases that were shorter in length and had increased branching (Figure 2B,C). This decrease in crease length and increase in branching could potentially drive the increased topographical density by facilitating the tighter packing of the surface features.

In the cell culture studies, the MSCs appeared to selectively adhere to the surface creases (Figure 3D, Figure 5A–C and Figure 6C–E). This phenomenon could be clearly visualised at a low seeding density, where a distinct alignment to the surface topographies could be observed and quantified. Moreover, live cell tracking data demonstrated that adhered cells actively migrated along the surface creases, avoiding cell–cell interactions in favour of maximising contact with the gel surface (Figure 3D). Surface topography has been widely reported to influence cell migration, directing cellular locomotion and adhesion [27,28,29,30,31]. The mechanism is yet to be fully elucidated, but it is postulated to involve cell contact guidance. Additionally, substrate stiffness and topography have previously been shown to impact protein orientation on the surface, which, in turn, affects cell behaviour [32]. In this instance, upon contact with the AZO-PA hydrogel surface, the MSCs likely receive topographical cues that promote cell polarisation, the extension of filopodia and lamellipodia, the alignment of the cytoskeleton and the subsequent focal adhesion to the surface features, as observed in previously reported studies on cell–matrix interactions [33,34,35,36]. Furthermore, the preferential adherence in the creases and avoidance of cell–cell interactions may shed light on the mechanisms of cell–matrix interactions, with MSCs potentially exhibiting a preference for binding to the fibronectin coating of the gel surface instead of binding to cell-synthesised binding molecules. In addition to this, it is possible that this system provides evidence that MSCs seek to maximise cell membrane contact and this drives the selective adherence within the creases, as they provide a pseudo three-dimensional extracellular environment.

Apart from directing cell migration along the surface creases, the gel features also influenced the cell morphology, promoting alignment and eccentricity (Figure 4 and Figure 5). Cells that adhered to the surface creases exhibited a significantly higher alignment and eccentricity than cells adhered to the spaces between. Multiple reported topographical cues, such as surface curvature and roughness, can stimulate such cellular functions, but, in this case, the cell body confinement was the most feasible driving mechanism. Adherence inside the confined spaces of the surface creases likely resulted in an increase in actin filament density. This forced the actin filaments to bundle together along a single axis and, subsequently, promoted cellular alignment along the length of the crease. This effect was consistent with previously reported data demonstrating that steric limitations on cell spreading, such as cell body confinement, can result in defined morphological changes, such as cell alignment [37,38]. Cells that adhered to the spaces between the surface features were not presented with the same spatial confinement, and, as a result, were able to spread more uniformly and with decreased alignment.

The topographical density and periodicity were also found to influence the morphology, but not cell size, with cells exhibiting reduced alignment in response to an increased topographic density. Gels produced with a higher topographic density developed shorter, less branched surface creases (Figure 2). Cells appeared to interact and be directly influenced by the topography. This reduction in crease size and branching likely caused the resulting changes in morphology, as cells were presented with less available surface area along which to polarise and align. Moreover, it is possible that in samples with a higher density of surface creases (e.g., Figure 2A, 50 mm gel thickness), the reduced distance between creases resulted in cells bridging gaps and adhering to multiple surface features. This may have inhibited adherence to a specific directionality, with cells anchoring to multiple surface features (Figure 5C).

Interestingly, while adherence to the surface topographies had no significant influence on the cell area (Figure 3), the dynamic modulation of matrix stiffness directly influenced the cell area, with the softened gel inducing a significantly reduced cell area and gel stiffening, causing a significant increase in area. Conversely, these changes in stiffness were found to have no effect on cell alignment or eccentricity (Figure 6). A reduction in matrix stiffness was also found to reduce the cell and nuclear size in a manner that was consistent with our previous study of MSCs cultured on AZO-PA, whereby softer gels gave smaller cell areas, even in the absence of surface features [19]. A clear trend was, therefore, observed, whereby the MSC shape and alignment could be regulated through changes in the topographical features of the AZO-PA gel, while the cell size could be directly altered through in situ changes in substrate stiffness.

## 5. Conclusions

Cell culture substrates have been repeatedly shown to influence cellular behaviour via mechanical and topographical cues. This study presented a method for tailoring the cell size and morphology using an AZO-PA hydrogel system with a tuneable stiffness and surface features. These textured hydrogels were easily prepared, and the creased surface features offered the cells a 3D-like environment without the need for complex 3D fabrication methods. The pseudo-3D surface that was generated with the gel fabrication was also convenient, as it could be studied using readily available microscopy equipment. Additionally, this system allowed for the decoupling of cell size from cell morphology, a capability that other materials do not currently facilitate. Overall, these results demonstrated the potential to utilise this material as a substrate for regulating and studying the mechanotransduction processes that regulate cell morphology, size and migration, and could help gain a better understanding of the interactions between cells and their extracellular environments.

## Data Availability

The data presented in this study are available on request from the corresponding author.
